# 

**Published:** 1888-10

**Authors:** 


CONTENTS:

ARTICLE I.—Some Affections of the Gums. By Frank Lankester, England .................... 241

" II.—Aluminum. By C. C. Carroll, M. D .................... 249

" III.—Joint Meeting of the American Dental Association and the Southern Dental Association. Reported by William Conard, D D. S., St. Louis, Mo .................... 252

" IV.—Perry's Remarks in Conjunction With Bonwill on Amalgams and Anticipation, etc. By Dr. W. G. A. Bonwill, Philadelphia, Pa .................... 258

" V.—Teeth with Dead Pulps, Without Fistule, and the Filling of roots. By Dr. J. Morgan Howe, New York ...... 262

" VI.—Dead and Diseased Teeth and their Treatment. By Dr. E. S. Niles .................... 269

" VII.—"Anæsthetics in Dental Practice." By J. C. Reeve, M. D., Dayton, Ohio .................... 273

EDITORIAL.

Correspondence Between F. J. S. Gorgas, Dean of University of Maryland Dental Department, and Dr. T. S. Waters, President of National Association of Dental Examiners .................... 278

What Cocaine to Use .................... 288

